# Investigating possible fraudulent activity at a research site

**DOI:** 10.1186/1745-6215-16-S2-P193

**Published:** 2015-11-16

**Authors:** Carol Knott, Joanne Henderson, Louise Bowman, Martin Landray

**Affiliations:** 1 Clinical Trial Service Unit, Nuffield Department of Population Health, University of Oxford, Oxford, UK

## Background

A risk-based approach to monitoring clinical trials aims to detect non-compliance with the protocol or regulatory requirements that may compromise the participants’ well-being or the trial’s ability to produce reliable results.

## Method

 We describe a pragmatic framework for investigating and evaluating suspicions of poor performance/practice and the steps to be taken if findings suggest fraudulent activity.

Prior to site audit:

• Check reliability of information that prompted concerns

• Prepare dossier of site information, detailing suspect data points

• Arrange visit promptly (but without indicating concerns)

During audit:

• Two auditors: one to ask open questions, the other as witness and scribe.

• Interview staff individually, establishing their knowledge of trial procedures and incident. Note conflicting information.

• Establish key facts:

1. Were participants real and eligible?

2. Did they consent?

3. Are test/clinical measurements/data valid?

4. Was appropriate treatment/intervention given?

5. Was follow-up provided, were events reported?

6. Are participants safe and data reliable?

• Obtain documentary evidence, maintaining confidentiality of trial participants

• Document findings

After audit:

• Implement corrective actions to:

1. Ensure safety of participants

2. Address important deficiencies in data quality

3. Support site: training, additional monitoring/support, replace staff.

• Look for systemic trial quality issues e.g. other staff, other data

1. Apply lessons/corrective actions to the whole trial

• Notify appropriate parties: Sponsor, steering committee, data monitoring committee, regulatory body, ethics board, host institution, funder, and professional bodies.

## Conclusion

Cases of serious misconduct or fraud do occur. Taking a systematic approach to an investigation ensures appropriate action is taken to preserve study intergrity.

